# Correction: An efficient multicomponent synthesis of 2,4,5-trisubstituted and 1,2,4,5-tetrasubstituted imidazoles catalyzed by a magnetic nanoparticle supported Lewis acidic deep eutectic solvent

**DOI:** 10.1039/d0ra90093a

**Published:** 2020-09-01

**Authors:** Thanh Thi Nguyen, Ngoc-Phuong Thi Le, The Thai Nguyen, Phuong Hoang Tran

**Affiliations:** Department of Organic Chemistry, Faculty of Chemistry, University of Science, Vietnam National University Ho Chi Minh City 721337 Vietnam thphuong@hcmus.edu.vn +84 903706762

## Abstract

Correction for ‘An efficient multicomponent synthesis of 2,4,5-trisubstituted and 1,2,4,5-tetrasubstituted imidazoles catalyzed by a magnetic nanoparticle supported Lewis acidic deep eutectic solvent’ by Thanh Thi Nguyen *et al.*, *RSC Adv.*, 2019, **9**, 38148–38153, DOI: 10.1039/C9RA08074K.

The authors apologise that a related reference, given here as ref. 1–5, was not cited in the original article. On page 38148, in the first paragraph of the Introduction, a citation to the reference should be added at the end of the sentence beginning “Among them, Lewis acidic…”. The paragraph should be changed as follows “In past decade, deep eutectic solvents (DESs) have attracted much attention in both reaction media and catalysts due to their unique properties such as wide liquid range, biodegradability, excellent thermal stability, and negligible vapor pressure.^1,2^ Among them, Lewis acidic deep eutectic solvents (LADESs) have been intensively studied as efficient media for organic syntheses.^3–5^”.

The Royal Society of Chemistry apologises for these errors and any consequent inconvenience to authors and readers.

## Supplementary Material
